# Recent Advances on Microbiota Involvement in the Pathogenesis of Autoimmunity

**DOI:** 10.3390/ijms20020283

**Published:** 2019-01-11

**Authors:** Elena Gianchecchi, Alessandra Fierabracci

**Affiliations:** 1 Infectivology and Clinical Trials Research Department, Children’s Hospital Bambino Gesù, Viale San Paolo 15, 00146 Rome, Italy; elegianche@yahoo.it; 2 VisMederi s.r.l., Strada del Petriccio e Belriguardo, 35, 53100 Siena, Italy

**Keywords:** microbiota, autoimmunity, etiopathogenesis

## Abstract

Autoimmune disorders derive from genetic, stochastic, and environmental factors that all together interact in genetically predisposed individuals. The impact of an imbalanced gut microbiome in the pathogenesis of autoimmunity has been suggested by an increasing amount of experimental evidence, both in animal models and humans. Several physiological mechanisms, including the establishment of immune homeostasis, are influenced by commensal microbiota in the gut. An altered microbiota composition produces effects in the gut immune system, including defective tolerance to food antigens, intestinal inflammation, and enhanced gut permeability. In particular, early findings reported differences in the intestinal microbiome of subjects affected by several autoimmune conditions, including prediabetes or overt disease compared to healthy individuals. The present review focuses on microbiota-host homeostasis, its alterations, factors that influence its composition, and putative involvement in the development of autoimmune disorders. In the light of the existing literature, future studies are necessary to clarify the role played by microbiota modifications in the processes that cause enhanced gut permeability and molecular mechanisms responsible for autoimmunity onset.

## 1. Introduction

The pathogenesis of autoimmune disorders is often multifactorial; in fact, in addition to a genetic predisposition [1,2,3], stochastic [4] and environmental [5,6] factors also play an important role in susceptible individuals. Among the environmental factors that have gained attention during the last decade, there is the intestinal microbiota community, which is involved in performing critical tasks for the normal development and maintenance of healthy human physiology.

A growing number of investigations have highlighted that humans are not born sterile, as we thought until some time ago; instead, intracellular bacteria have been identified even in the placenta, amniotic fluid, umbilical cord, and meconium [7]. The gastrointestinal lumen, representing the widest surface area in the human body exposed to environmental factors and in contact with a high number of different antigens and microbes [8], becomes inhabited by bacteria, viruses [9], *Archaea* [10], and fungi immediately after birth [11]. *Actinobacteria*, *Bacteroidetes*, *Proteobacteria*, and *Firmicutes* are the most abundant bacterial phyla in the mouse and human intestine during homeostasis. The colonization of the gut does not represent a random event, but is the result of the evolutionary process as demonstrated by the fact that microbiome composition of humans and other mammals presents an elevated level of conservation with respect to the same phylum level [12]. The colonization of the gut by microbiota represents a dynamic, complex, and gradual process, which is also in continuous development in the early years of life in parallel with the development of the immune system of the newborn.

Distinct composition and diversity of the gut microbiome occurs even during pregnancy, along with physiologic, metabolic, and immune variations of the woman [13]. The recent study of Gomez de Agüero [14] suggests that early postnatal innate immune development could be critically influenced by maternal microbiota transfer, as well as that of its metabolites. In addition, birth delivery mode, breastfeeding, and food introduction constitute some of the factors able to influence the microbiome in the newborn. More in detail, the birth delivery mode seems to be able to drive the diversity of the infant gut microbiome, although conflicting results are reported about the putative correlation between birth delivery mode and the risk of developing non-communicable diseases later in life [15].

Indeed, when natural delivery occurs, contact with vaginal and fecal microbiota of the mother is favored, while during the caesarian section (CS), contact with commensal bacteria on other surfaces, such as the skin is promoted, avoiding or dramatically reducing contact with maternal vaginal and fecal microbiota [16]. CS was not only associated with a reduced microbiome diversity, with respect to natural delivery [17], which persisted until 7 years of age [18], but was also correlated with diminished Th1 chemokines in the blood [18] and with an increased risk to develop childhood T1D [17,19].

The crosstalk between the microbiome and the host is fundamental since it induces the proper gut epithelial construction and activity, as well as metabolism and nutrition. The gut epithelial barrier, constituted by a single cell layer, represents the interface between the host and microbiota, allowing metabolites to access and interact with host cells. On the other hand, maintenance of gut integrity is fundamental since impairment of the gut and mucosal barrier could allow microbes to enter the *lamina propria* and systemic blood circulation inducing an imbalance in the host immune homeostasis and leading to systemic immune hyperactivation.

Several functions can be exploited only upon bacterial activity. More in detail, these include the metabolism of complex glycans, amino acids, and xenobiotic, and the synthesis of short-chain fatty acids (SCFAs) and vitamins [16]. Moreover, the microbiome is able to impede pathogens, such as *Shigella flexneri* and *Salmonella*, that could enter host cells, preventing development of inflammation promoted by dysbiosis. An inflammatory condition can be elicited by microbes identified as pathobionts in host harboring an aberrant microbiota caused by the use of antibiotics or in genetically predisposed individuals [11].

If, on one side, the host immune system controls microbial ecology, on the other side, microbiota produces a wide variety of biochemically active compounds, such as neurotransmitters and tryptophan-derived metabolites [20,21] influencing both the maturation and activity of the immune system. Given the importance of a proper regulation of host immune response, increasing interest in the wide microbial metabolite repertoire has emerged. These metabolites represent compounds produced by the host or by the microbiome itself (such as polyamines), or are derived from the bacterial metabolism of substances introduced with the diet. Among the latter are found ligands of the aryl hydrocarbon receptor (AHR), polyamines, and SCFAs, which encompasses butyric acid, acetic acid, and propionic acid, and that originate from undigested complex carbohydrates [22].

AHR signaling is fundamental for the maintenance of host immune homeostasis. Indeed, the protective role of AHR ligands has been demonstrated against the fungal pathogen *Candida* (*C.*) *albicans* and against mucosal inflammation through the IL-22 synthesis [23].

Polyamines reinforce the intestinal epithelial cell barrier by inducing the synthesis of intercellular junction proteins, such as E-cadherin, *zonula occludens* 1, and occludin [24]. Furthermore, they play a role in host immunity through the inhibition of macrophage activation and pro-inflammatory cytokine synthesis. In addition, they can modulate mucosal and systemic adaptive immunity [22].

SCFAs exert several functions affecting host physiology: they represent a considerable source of energy for intestinal epithelial cells, induce mucin gene transcription [25], and influence the permeability of tight junctions. As a consequence, the epithelial barrier is strengthened, thus preventing toxic compounds from entering the blood stream. In addition, as AHR activation, SCFAs also have an effect on host immunity. Indeed, the tolerogenic phenotype and thus immune homeostasis, as well as T regulatory cell (Treg) development, are promoted by SCFAs through the inhibition of histone deacetylases [26].

The majority of intercellular signals is mediated by bioactive molecules, such as lipopolysaccharide (LPS), proteins, toxic and immunomodulatory mediators, RNA or DNA, and ATP [27] contained into secreted extracellular vesicles (EVs) [28]. EVs are spherical particles that are membrane-bounded and characterized by a diameter of 50–250 nm. This protects their content from nucleases and proteases, and properly delivers it to neighboring or distant cells [29]. EVs can be secreted by eukaryotic cells, and Gram-negative [28] and Gram-positive bacteria [29].

The symbiotic interaction between the human host and microbiome is so strict that the microbiome has also been defined as a prokaryotic organ with complex endocrine functions [26]. Increasing evidence has reported that alterations in the composition and/or abundance of the gut microbiome and subsequent alterations in its metabolic network correlated with the onset and even the progression of various intestinal and systemic autoimmune conditions. Nevertheless, the mechanisms underlying the dysregulation of gut microbiota and autoimmunity remain to be elucidated.

## 2. Microbiome Composition and Autoimmune Conditions

Recently, increasing interest has been directed towards the identification of the gut microbiome, the principal source of microbes, and the understanding of its positive or negative effects on health of human beings, in particular in the light of the recent evidence that the inflammatory process could be elicited by the microbiome.

A growing number of studies have supported the striking linking of altered microbiota composition with the onset of several different autoimmune disorders (Table 1). These include Type I diabetes (T1D) [16,30,31,32,33,34,35,36,37,38,39,40,41,42,43,44,45,46,47,48,49,50,51,52,53]; rheumatoid arthritis (RA) [54,55,56,57,58,59,60,61,62]; systemic lupus erythematosus (SLE) [63,64,65,66,67]; inflammatory bowel disease (IBD) encompassing Crohn’s disease (CD) and ulcerative colitis (UC) [68,69,70,71,72,73,74,75,76,77,78]; Behcet’s disease (BD) [79,80,81,82]; autoimmune skin conditions including vitiligo [83], psoriasis vulgaris [84,85,86,87,88], and atopic dermatitis [89,90,91,92,93,94,95,96,97]; and autoimmune neurological diseases [98,99,100,101,102,103,104,105,106]. Several alterations involving microbiome have been identified in autoimmune patients and thus hypothesized to have a role in the onset of the different autoimmune conditions (Table 2).

### 2.1. Type 1 Diabetes

Type 1 diabetes (T1D) is an organ-specific autoimmune condition characterized by the specific destruction of pancreatic β cells of the islet of Langerhans deputed to insulin release. This destructive process is operated by cytotoxic T lymphocytes (Tc) [139]. As a consequence, glucose cannot be absorbed by cells. The insulitis lesion is characterized by several infiltrating cells, such as B and T lymphocytes. T-helper 1 (Th1) lymphocytes are responsible for the pancreatic infiltration and for the sustenance of cytotoxic T (Tc) lymphocyte activity by secreting cytokines [140]. Millions of people are affected by T1D worldwide, whose etiology is multifactorial [141]. In more detail, it has been suggested that a combination of environmental, genetic, and stochastic factors are responsible for its pathogenesis [142,143]. T1D incidence has been steadily rising during the last 50 years, probably due to modifications in the gut microbiota associated with modern lifestyles, which may be responsible for a defective development of the immune system.

Recent investigations conducted on animal models [30,31,32,33,34,38,45] and humans [36,37,39,40,42,43,47] have implicated a causal role for the alterations in the normal flora in T1D development [41] through an upsurge in gut permeability [115,116,117,118]. The shifts in the intestinal microbial populations before T1D clinical onset support the role played by gut bacteria in T1D etiopathogenesis. The identification of these changes is fundamental to understand the events implicated in the progression of the disease, even though limited knowledge regarding bacterial functions involved in T1D is available yet.

Bosi et al. [119] observed that T1D onset was preceded by enhanced intestinal permeability.

Non-obese diabetic (NOD) mice carried a “diabetes-permissive” microbiota different from that harbored by T1D-protected inbred strains. Furthermore, important imbalances affecting microbiota can both reduce and accelerate T1D onset [144].

T1D incidence was increased when NOD mice were grown in a specific-pathogen-free (SPF) environment or clean housing facilities compared to conventional conditions [33] and changes perturbing gut microbiome, like diet modifications, can prevent disease [145]. Also, antibiotic treatment accelerated T1D onset in mice by substantially shifting the gut microbiota [120,121,122], and this was particularly important in the case of early life antibiotics exposure [121]. Roesch et al. [146] found a higher *Bifidobacter*/*Lactobacilli* ratio in the diabetic-resistant rat group with respect to the diabetic sensitive rat group. Sun et al. [45] demonstrated that changes in gut microbiome limited autoimmune diabetes in NOD mice by synthetizing an immunoregulatory cathelicidin-related antimicrobial peptide in islets.

High-risk children presenting islet-autoantibodies had distinct bacterial diversity with respect to low-risk autoantibody-negative children [35,36]. Kostic et al. [43] reported a reduced bacterial diversity prior to the onset of clinical disease in an investigation conducted on high-risk Finnish children; these data were in accordance with those reported by Alkanani [42] demonstrating the correlation between T1D susceptibility and intestinal microbiome perturbations in a U.S.-based cohort.

Most of the studies assessing impairment of the gut microbiome balance and T1D have been conducted on Caucasians; however, the incidence of the disorder was quickly rising during the past decade in China, allowing us to speculate the role of non-genetic factors. Therefore, Huang et al. [51] have evaluated gut microbiota profiling in 12 T1D Han Chinese and 10 healthy controls by using 16S rRNA sequencing followed by analyses of the gut microbiota composition. The study proved important differences in 28 bacterial taxa (13 increased and 15 decreased) in T1D patients as compared to controls. In T1D subjects and controls, *Bacteroidetes* and *Firmicutes* constituted the dominant phyla, respectively. The raised *Bacteroidetes*/*Firmicutes* ratio was in accordance with a previous study conducted on Caucasians T1D and high-risk cohorts [44]. Also, Murri [37] reported an increase in *Bacteroides* in Spanish T1D children respect to controls along with a higher abundance of *Clostridium* and *Veillonella* and a reduction in the populations of *Bifidobacterium*, *Lactobacillus*, the *Blautia coccoides*/*Eubacterium rectale* group, and *Prevotella*.

Likewise, the Pinto [47] group reported the rise in *Bacteroides* and *Clostridium* cluster XVa and cluster IV concurrently with a reduction in *Bifidobacterium*. The proteome analysis distributed the most representative bacterial proteins in functional groups thus revealing marked differences between Portuguese T1D children and controls. In fact, whereas in the latter most of the proteins correlated with metabolism and transport of carbohydrates, in the former group, among the most abundant proteins some were specific for T1D, such as those involved in transport and metabolism of amino acids and coenzymes, meaning that a functional enrichment in core energy metabolism proteins, in particular on sugar transport and processing is involved.

It has been speculated that a *Bacteroides* expansion could affect the integrity of epithelial tight junctions and halt Treg differentiation by the products, such as acetate and succinate, generated with the anaerobic respiration [44]. The recent intestinal metaproteomics study conducted by Gavin [48] on 33 subjects with recent-onset T1D, 17 islet autoantibody-positive subjects, 29 low-risk autoantibody-negative subjects, and 22 healthy individuals revealed the presence of specific host-microbiota interactions in T1D patients presenting considerable alterations in the prevalence of host proteins correlated with exocrine pancreas output, inflammation, and mucosal function. In addition, T1D subjects showed a higher intestinal inflammation and reduced barrier activity due to a depletion in microbiota taxa related with host proteins implicated in the maintenance of microvilli adhesion, mucous barrier, and exocrine pancreas functionality.

The evaluation of the possible correlation between autoantibodies and bacterial abundances revealed a positive association between anti-islet cell autoantibodies with *Bacteriodes* and *Bilophila*, while a negative association was found with *Streptococcus* and *Ruminococcaceae*. In addition, *Faecalibacterium* abundance was negatively correlated with levels of glycated hemoglobin A1c (HbA1c) [51], in contrast with the study of Hornef [147] reporting similarity in the phylum level between T1D Han Chinese children and healthy controls.

Recently, Gursoy et al. [49] have investigated the potential role played by the intestinal colonization of the opportunistic fungus *C. albicans*, which is part of the normal intestinal microflora, on T1D onset and gut integrity. Intestinal *C. albicans* colonization was found in 50% of T1D patients and 23.8% of controls at the time of diagnosis in a total of 42 newly-diagnosed T1D patients and 42 healthy controls. These findings support the hypothesis that the autoimmune response, like that responsible for T1D onset, could be due to alterations in the normal gut microbiome composition by enhancing intestinal permeability. Soyucen et al. [41] analyzed the fecal flora of 35 newly diagnosed Turkish T1D patients and 35 healthy subjects reporting a significant decrease in *Bifidobacterium* colonization, in accordance with de Goffau et al. [36], and a considerable increase in *C. albicans* and *Enterobacteriaceae* in T1D patients was found with respect to controls. Regarding *Escherichia* (*E.*) *coli*, no differences between the two groups were observed. Although a reduction in *Bacteroides* spp. and *Lactobacillus* colonization characterized T1D subjects with respect to controls, these differences did not reach significance. However, it has been supposed that the higher *C. albicans* colonization could be due to the reduction of *Lactobacillus*, *Bifidobacterium*, and *Bacteroides* species. Indeed, they constitute beneficial anaerobic bacteria within the gut since they exert an inhibitory function by synthetizing short-chain fatty acids and antimicrobial compounds. These alterations, besides *Enterobacteriaceae* and the slight and non-significative *E. coli* increase observed in the T1D group, could lead to islet destruction as a final step of the autoimmune process.

Although more data are necessary to fully understand the correlation between β islet destruction and the potential causal role of microbiome and diet, their interaction is critical, as demonstrated by the correlation between early probiotic supplementation and diminished risk of islet autoimmunity in children with a high genetic risk for T1D [46].

Recently, Henschel [50] reported that the peripheral inflammatory state correlated with autoimmune diabetes susceptibility was kept under control following modulation of the diet and gastrointestinal microbiota. More specific, spontaneous diabetic BB DR*lyp*/*lyp* and diabetes inducible BB DR^+^/^+^ weanings fed with a standard cereal diet presented a considerable pro-inflammatory transcriptional expression consistent with microbial antigen exposure. This inherent inflammatory state (i.e., the presence of the pro-inflammatory islet transcriptome as well as β-cell chemokine expression) was reverted when DR^+^/^+^ weanings were fed with a gluten-free hydrolyzed casein diet (HCD) or treated with antibiotics and reduced T1D incidence was proven in lymphopenic DR*lyp*/*lyp* rats. Moreover, the introduction of gluten to HCD reverted these effects. The sequencing of bacterial 16S rRNA gene highlighted that diet changes or antibiotic treatments disrupt ileal and cecal microbiota, with an enhancement in *Firmicutes*/*Bacteriodetes* ratio and in the relative abundances of lactobacilli and butyrate producing taxa [50]. A recent study of Mullaney et al. [52] found that specific gut microbial imbalance were linked to T1D susceptibility alleles in mice. Accordingly, immune tolerance towards islet antigens was re-established upon disease protective allele introduction which induced a restoration of the gut immune regulatory system and microbiome modifications. Additional studies demonstrate bacterial metabolites can affect host immune system and induce beta cell autoimmunity and T1D [53].

### 2.2. Rheumatoid Arthritis

Rheumatoid arthritis (RA) constitutes a systemic inflammatory chronic disorder wherein joints, representing the target of the inflammatory process, undergo destruction. In addition to joints, other organs can also be affected by the autoimmune process, such as the lungs and the gastrointestinal tract. The etiopathogenesis is multifactorial: environmental, genetic, hormonal, and immunological factors have been hypothesized to have a role in RA onset [148,149].

The involvement of gut microbiome in RA pathogenesis is supported by the observation that germ-free mice were protected against experimental arthritis [123].

Although segmented filamentous bacteria (SFB) constitute a smaller though important component of the commensal flora, its ability to shape the immune response of the host by inducing intestinal T helper 17 (Th17) lymphocytes [124] has been demonstrated. It was shown that autoimmune arthritis was caused by gut-residing SFB via Th17 cells [123,125], which, in association with a T follicular helper (Tfh), promoted autoantibodies in young K/BxN mice [125].

In accordance with these observations, interleukin-17 (IL-17) neutralization inhibited AR onset [150] and a substantial amelioration in the production of autoantibodies, as well as in autoimmune arthritis development that occurred when Tfh and Th17 cells were lost [125]. It has been thought that Th17 compartment carrying dual T cell receptors (TCRs) recognizing both microbial and self-antigens may be specifically expanded by SFB causing lung autoimmunity onset, an important extra-articular RA manifestation and the principal cause of RA-related mortality. In detail, whereas during the pre-arthritic phase SFB promoted autoantibodies, the RA-related lung pathological changes were due to Th17 recruitment as a consequence of the strong chemokine (C-C motif) ligand 20 (CCL20) expression, representing a chemoattractant for Th17 in this organ [126].

A perturbation of the gut microbiome in RA patients has been reported [56,107] with a higher abundance of the pathobiont *Prevotella* (*P. copri*) in new-onset RA subjects, while the reduction observed in established RA subjects could be due to the treatment [107]. The role of this bacterium is also sustained by the study of Pianta [58], who found molecular mimicry between its epitopes and two autoantigens, *N*-acetylglucosamine-6-sulfatase (GNS) and filamin A (FLNA). In addition to gut dysbiosis, the microbiome of the dental region and saliva were also altered [56]. In particular, an association between periodontal infections due to *Porphyromonas* (*P.*) *gingivalis* and RA have been envisaged [54,108]. This hypothesis is supported by both the correlation between oral and gut flora and the higher incidence of periodontitis in RA subjects [108] and by a recent investigation conducted on animal model of RA demonstrating worsening of collagen-induced arthritis (CIA) upon the oral administration of *P. gingivalis* [59] through the enhanced synthesis of IL-17 [55].

The research conducted by Teng [60] using young and middle-aged K/BxN T cells aimed to investigate the impact exerted by age and microbiome on autoimmune arthritis, since this pathology usually develops in middle age, whereas most of the studies conducted until now were performed on young adult mice. When compared with younger mice, the old counterparts with the same TCR specificity presented a higher number of Tfh, representing a reminiscence of those differentiated during the early age that have survived during the aging process. Tfh had an effector memory phenotype (CD62L^lo^CD44^hi^) in the majority of the cases. Tfh presented a low response when antigen was newly introduced in middle age, but they recognized the self-antigen from youth as occurring in RA during the latent phase [151]. This alteration was associated with a significantly impaired Th17 response not due to a defective Th17 proliferation but to an inefficiency of middle-aged autoimmune CD4^+^ T cells to differentiate into Th17. In addition, a reduced IL-23 expression level was found, which is fundamental for the maintenance of Th17. Likewise, a lower differentiation of IL-17-producing cells from naive CD4^+^ T cells in older mice with respect to the younger group was observed, resembling the observation obtained in humans [152,153]. The discrepancies concerning Th17 number were probably due to the age of mice used in the two studies [60]. The investigation of the contribution of age on AR is particularly important since it represents a risk factor for AR.

A correlation between dysbiosis of the oral microbiome, periodontitis, and the production of citrullinated proteins was also observed in RA patients. Indeed, among the autoantibody profile characterizing RA patients, there are antibodies recognizing anti-citrullinated products that could represent novel epitopes upon being targeted by the post-transcriptional modification of citrullination. According to this hypothesis, a recent study of Pianta [58] reported the identification of two self-antigens FLNA and GNS recognized by B and T lymphocytes in RA subjects. More in detail, GNS was citrullinated, and GNS antibody values correlated with anti–citrullinated protein antibody (ACPA) levels. Not only were FLNA and GNS both greatly expressed in inflamed synovial tissue, but their T cell epitopes presented also homology with *Prevotella*, *Parabacteroides sp*., *Butyricimonas sp.*, and other gut microbes [58]. This evidence has allowed us to speculate the putative role of citrullinated products as a self-antigen in subjects with a genetic predisposition for RA [154]. Along with citrullinated proteins, proinflammatory cytokines have also been hypothesized to be implicated in the linkage of RA with periodontal disease as witnessed by the rapid RA development in the adjuvant arthritis model in case of pre-existing extra-synovial inflammation due to *P*. *gingivalis* [155].

Recent studies have in fact highlighted the role played by the peptidylarginine deiminase (PAD) enzyme derived from *P. gingivalis* and *Aggregatibacter* (*A.*) *actinomycetemcomitans*, which citrullinates human fibrinogen, alpha-enolase [127], and peptides from critical RA autoantigens [128] and causes hypercitrullination in the rheumatoid joint in host neutrophils [129].

As reported for other autoimmune conditions, in RA subjects, several alterations in gut microbiome with respect to healthy individuals have also been reported [57,61,62]. A stronger reduction in gut microbial diversity was proven in RA patients with respect to controls, and it was related to disease duration and autoantibodies levels. Furthermore, Chen et al. observed a decrease in abundant taxa accompanied by an expansion of rare lineage intestinal microbes in RA patients respect to controls [57].

Picchianti et al. [62] noted substantial changes involving mainly lower taxonomic levels, while the relative abundance of the microbial phyla was not modified. More specifically, *Bacilli* and *Lactobacillales* were enhanced, while the genus *Faecalibacterium* and the species *Faecalibacterium* (*F.*) *prausnitzii* were importantly decreased in RA patients with respect to controls. Moreover, the latter presented the genus *Flavobacterium* and the species *Blautia* (*B.*) *coccoides*, which have not been observed in RA subjects [62].

### 2.3. Systemic Lupus Erythematosus

Systemic lupus erythematosus (SLE) is a systemic autoimmune disease characterized by B cell hyperactivity and the presence of several circulating autoantibodies [156,157], with a higher incidence in women. Several factors, such as genetic and environmental factors, drugs, infections, and immune system defects (reviewed in Reference [157]), seem to contribute to SLE etiology. In addition to the investigation of the putative correlation between SLE and the human microbiota [65,158], the role of periodontal disease in SLE condition [63,64,66,159] has been furthered since, as demonstrated in RA (*vide supra*), periodontal pathology may aggravate SLE severity by increasing systemic inflammation. Furthermore, a limited number of studies with opposite results, partially attributed to small sample size, are available so far.

Recently, Corrêa and colleagues [67] investigated for the first time the influence of SLE on the oral microbiota on 52 SLE patients and 52 healthy subjects finding a positive correlation between SLE and periodontal disease that affected 67% of SLE patients. Moreover, SLE patients showed a dysbiotic subgingival microbiota, with a more elevated subgingival bacterial load and a reduced microbial diversity at the diseased sites than controls. A dysbiotic condition correlated with an increased inflammation, as revealed by higher levels of inflammatory cytokines (IL-6, IL-17, and IL-33) in SLE subjects with periodontitis, is in accordance with the finding of Mendonça [109]. Independently from periodontal status, SLE patients presented an expansion of bacterial species frequently characterizing periodontitis, including *Fretibacterium*, *Selenomonas*, and *Prevotella* (*P*.) *nigrescens*; the latter has also been found to be increased in RA [55]. In addition, the analysis of the subgingival microbiota collected from SLE patients and healthy subjects revealed that the microbiome was influenced by SLE since they were characterized by a shift toward greater proportions of pathogenic bacteria. This was in accordance with previous data [110] reporting a positive association among periodontal pathogens (*Treponema denticola*, *P. gingivalis*, *Fretibacterium fastidiosum*, and *Tannerella forsythia*). A higher periodontal damage or inflammatory response favoring periodontitis could be promoted via dysbiosis of the subgingival microbiota; on the other hand, severity of SLE could be worsened by the presence of a periodontal condition as demonstrated by the fact that periodontal inflammation correlated with more severe SLE scores [67]. In light of these observations, the importance of a strict monitoring of dental health status of SLE patients is evident, and eventually treating periodontal inflammation during the starting phase becomes urgent.

### 2.4. Behcet’s Disease

Behcet’s disease (BD) represents a chronic multisystemic inflammatory condition characterized by the presence of uveitis, skin lesions, recurrent oral aphthous, and genital ulcers [160,161], and can involve the gastrointestinal tract (intestinal BD) [160,161,162] and the central nervous system (CNS) [150,161]. Importantly, it represents one of the principal causes of blindness. IBD etiology is caused by environmental and genetic factors [150,161,163], including microbial factors in genetically susceptible individuals [150,160,164]. BD patients show defects in Th1, Th17, and Treg cell functions [165,166], which have been demonstrated to be regulated by the gut microbiome [130,131]. The possible association between BD and specific changes in the gut microbial community has been showed [79,80,81].

The presence of marked modifications in BD conditions has been recently confirmed by the metagenomic study conducted by Ye et al. [82] analyzing fecal and saliva samples collected from 32 active BD patients and 74 healthy controls. *Bilophila* spp. and several opportunistic pathogens (e.g., *Parabacteroides* spp. and *Paraprevotella* spp.) resulted in an increase in fecal samples from active BD patients, whereas a reduction was observed in butyrate-producing bacteria (BPB) *Clostridium* spp. and methanogens (*Methanoculleus* spp. and *Methanomethylophilus* spp.). These changes were associated with altered biological microbial functions with an enhanced oxidation–reduction process, capsular polysaccharide transport system, and type III and IV secretion systems. Accordingly, the fecal microbiota transplant from active BD patients in B10RIII mice strongly exacerbated experimental autoimmune uveitis (EAU) activity with strong inflammatory cell infiltration within the retina, the choroid, and the vitreous cavity. Conversely, healthy control-recipient mice and PBS-treated group showed merely a weak intraocular inflammatory reaction. Moreover BD-recipient mice had an enhanced inflammatory cytokine synthesis of IL-17 and interferon gamma (IFN-γ) with respect to the two control groups [82].

### 2.5. Inflammatory Bowel Disease

IBD encompasses Crohn’s disease (CD) and ulcerative colitis (UC). IBD is a complex disorder in which a chronic inflammation affects the gastrointestinal tract with frequent extra-intestinal manifestations [167]. A combination of non-genetic and genetic factors could be responsible for IBD pathogenesis, although its etiology remains to be elucidated [168]. The disease is supposed to be due to altered innate and adaptive immune responses directed towards pathogen associated molecular patterns (PAMPs) derived from microorganisms constituting the intestinal flora in genetically susceptible individuals. A role for the intestinal microbial community in the onset and chronicity of CD is strongly suspected. However, investigation of such a complex ecosystem is difficult, even with culture-independent molecular approaches.

An impaired epithelial barrier and increased intestinal permeability observed in UC and CD patients sustain this hypothesis [167]. A correlation between IBD and a decrease in gut bacterial diversity upon an imbalance from commensal in favor of potentially pathogenic species (identified as “dysbiosis”) has been reported [68,70,71,77]. In particular, recent data are supporting the critical role of cellular stress signaling involving the gut microbiome in the mucosa of the gastrointestinal tract. The microbiome fosters intestinal health and at the same time could have a role in IBD onset through complex interactions with the stress signaling pathways in host cells [78].

In the metagenomic study performed by Manichanh et al. [70], a full range of intestinal microbes were investigated by using two libraries of genomic DNA isolated from fecal samples obtained from six CD patients and six healthy donors. The study revealed a diminished diversity in the bacterial phylum *Firmicutes* (*F.*) *faecal* in the microbiota of CD subjects. In more detail, a significant reduction of the *Clostridium leptum* phylogenetic group was reported in CD patients compared to healthy subjects. In addition, novel bacterial species were observed [70].

Frequently, an enhancement in the *Proteobacteria* phylum including *E. coli* has been observed in patients with UC or CD, whereas *Firmicutes* phylum was reduced in the fecal samples of CD patients respect to healthy individuals [69,70,73].

Takahashi [76] found a decline in several butyrate-producing bacteria species in the fecal microbiome of CD patients in accordance with a previous study performed by Wang [74]. More specifically, a meaningful reduced abundance of the genera *Bacteroides*, *Eubacterium*, *Faecalibacterium*, and *Ruminococcus*, and increased proportion of the genera *Actinomyces* and *Bifidobacterium* were reported in CD patients respect to healthy controls. At the species level, a considerable reduction of butyrate-producing bacterial species, as *Blautia faecis*, *Roseburia inulinivorans*, *Ruminococcus torques*, *Clostridium lavalense*, *Bacteroides uniformis*, and *F. prausnitzii* characterized CD patients respect to healthy subjects. Similar results were observed in further CD patients (*n* = 68) and healthy controls (*n* = 46) [76].

The presence of important differences in mucosa-associated gut microbiota have also been observed in children affected by IBD [112,169,170]. Dysbiosis could be present before CD onset, as demonstrated by the group of Gevers, who found disturbances in the microbiota composition of the stool and mucosal in newly diagnosed, treatment-naive children affected by CD [171]. IBD subjects also showed alterations in the composition of bacteriophages with an increase in *Caudovirales bacteriophages* [113], as well as in fungal composition. Alterations in the diversity of the latter characterized mucosa and fecal samples. Even though the exact role of fungi in IBD development has not been clarified, host metabolism and mucosal immune response, as well as the microbiome composition, and thus the homeostasis of the gut more in general, could be influenced by fungi as supported by animal studies [172]. Indeed, even fungi and viruses constitutes the microbiome of the gut, with the predominance of bacteriophages as demonstrated by metagenomic analyses executed on viral particles from human stool samples [173,174]. Recently, Van Belleghem [175] observed that *Staphylococcus* (*S.*) *aureus* and *Pseudomonas aeruginosa* phages exert immunomodulatory activities on human peripheral mononuclear cells, whereas a limited number of studies have investigated the role of bacteriophages in IBD pathogenesis [176]. Concerning the contribution of diet and metabolism to IBD pathogenesis, aryl hydrocarbon receptor (AHR) agonists seem to play a role in several autoimmune conditions, including IBD by modulating T cell responses. Intriguingly, reduced levels of AhR expression agonists characterized the inflamed intestinal tissue samples collected from CD respect to uninvolved areas of the same patients, and UC and control subjects. The anti-inflammatory effect exerted by the Ahr agonist on the gastrointestinal tract was mediated by the induction of IL-22 [177], accordingly with previous studies reporting the anti-inflammatory [178] and protective [132] role of this cytokine. On the light of these results, AhR-related molecules could represent a promising treatment for CD.

### 2.6. Autoimmune Skin Conditions

#### 2.6.1. Vitiligo

Vitiligo is a chronic pigmentary disorder affecting 1% of the population. It represents an acquired depigmentary skin disorder leading to the development of white macules that are caused by a reduction of the number and function of melanocytes in the skin and/or hair [133]. The pathology presents a systemic involvement since melanocytes are located not only in the skin but also on various parts of the body [134]. Its origin remains to be elucidated and among the various hypothesis [179,180], the main one is an autoimmune attack of melanocytes. Furthermore, it is frequently associated with other autoimmune diseases [181]. The available treatment aims to reduce the exaggerated immune reaction, although with limited positive results.

The group of Ganju [83] investigated the differences in bacterial community of lesional and non-lesional skin of vitiligo subjects. The analysis of community composition revealed that four phyla (*Actinobacteria*, *Proteobacteria*, *Firmicutes*, and *Bacteroidetes*) dominated in both skin types, in accordance with previous data of microbiota composition of healthy skin [114]. However, they highlighted the presence of dysbiosis in the diversity of the microbial community structure in the lesional skin of vitiligo subjects with a reduction in taxonomic richness. Furthermore, they evaluated the presence of networks between individual microbiota members through intra-community network analysis investigating various network properties (which includes nodes, edges, density, diameter, etc.), and centrality measures (degree and betweenness). The study allowed for the reveal of a specific pattern of interactions between resident bacterial populations of the two sites (lesional and non-lesional). Lesional skin areas present an aberrant intra-community network since bacteria had a reduced number of interactions respect to those of non-lesional sites. In more detail, *Actinobacterial* sp. and *Firmicutes* prevailed in the central regulatory nodes of non-lesional skin and in lesional sites, respectively. Although the dynamics characterizing the bacteria constituting the cutaneous microbiome remains to be elucidated, the alterations observed in the microbiome composition of vitiligo lesions allowed researchers to envisage their implication in the persistence and the severity of vitiligo. If such a hypothesis will be confirmed, skin bacterial populations could represent a valuable target for vitiligo treatment.

#### 2.6.2. Psoriasis Vulgaris

Psoriasis vulgaris represents a common chronic inflammatory skin disease caused by iper-activated immune pathways of both the innate and adaptive immunity that in normal conditions are constitutive or inducible [182]. The common type of psoriasis is also denominated by large plaque psoriasis or psoriasis vulgaris and is characterized by red colored plaques with well-defined borders and silvery-white dry scale, involving elbows, knees, scalp, and the lumbosacral area. However, psoriasis lesions can also be more extensive. Besides this form of psoriasis, there are also further variants of the disease: guttate, inverse, pustular, erythrodermic, palmo-plantar, and drug-associated psoriasis [135,183,184]. The possible correlation between the disease and the skin microbiome has been investigated by a small number of studies. The existing contrasting results can be caused by the absence of standardized sampling and protocols, or to an intrinsic variability of microbes among humans [84,85,86,87,185].

The study performed by the group of Alekseyenko [86] focused on the characterization of skin microbiota of psoriatic lesions, unaffected contralateral skin from 75 chronic plaque psoriatic patients, and similar skin loci in 124 matched healthy subjects through high-throughput 16S rRNA gene sequencing. Psoriasis was characterized by physiological alterations, both at the lesion site and at the systemic level, which were able to modulate microbiome composition among the clinical skin types evaluated. More specifically, a reduction in the taxonomic diversity as well as in the evenness in both lesion and unaffected microbiota communities from psoriatic patients with respect to the control was observed. The analysis of the relative abundance of the taxa constituting the skin microbiota revealed that three phyla *Proteobacteria*, *Firmicutes*, and *Actinobacteria* prevailed in the skin microbial communities in all three subgroups, according to previous studies [85]. Furthermore, psoriasis correlated with relative abundance and presence of specific taxa. More in detail, even though the three subgroups evaluated (lesion, unaffected, and control) did not present significant differences in the genera usually present on skin, i.e., *Propionibacterium*, *Corynebacterium*, *Streptococcus*, and *Staphylococcus*; they were characterized by considerable differences in the combined relative abundance of the four taxa. *Corynebacterium*, *Propionibacterium*, *Staphylococcus*, and *Streptococcus* showed a higher abundance, whereas a marked reduction in relative abundances of *Cupriavidus*, *Flavisolibacter*, *Methylobacterium*, and *Schlegelella* genera were observed in psoriatic patients with respect to controls. The study highlighted the association between lesion samples with *Firmicutes*-associated microbiota. A recent investigation conducted by Chang [88] confirmed alterations affecting skin microbiome in psoriasis, and further analysis conducted on mice colonized with *S. aureus* demonstrated a marked upregulation of Th17 response, which could have a role in IL-17-driven inflammation in psoriasis. Accordingly, mice colonized with *Staphylococcus epidermidis* or un-colonized mice (controls) did not present this response.

#### 2.6.3. Atopic Dermatitis

Atopic dermatitis (AD) represents a chronic recurrent inflammatory cutaneous disease whose patients present itching and xerosis [186]. AD is also associated with other allergic diseases. An enhancement of its prevalence has been observed in developed countries, affecting from 15% to 30% and 2% to 10% of children and adults, respectively [187]. The pathology is in fact the skin manifestation of a systemic disorder in which both local and systemic factors are implicated in its etiology. AD skin is characterized by dysbiosis with marked *S. aureus* colonization [94], which has also been positively associated with disease severity.

The investigation conducted by Laborel-Préneron [91] revealed the correlation between *S. aureus* in inflamed skin of AD subjects and elevated IgE response and up-regulation of inflammatory and Th2/Th22 transcripts. Furthermore, secretomes from *S. aureus* and *S. epidermidis* from the skin microbiota of AD children induced monocyte-derived dendritic cells to produce pro-inflammatory IFN-γ and anti-inflammatory IL-10, respectively. *S. aureus* and *S. epidermidis* secretomes also exerted the opposite effect on CD4^+^ T cell activation, which was induced by the former, whereas CD4^+^ proliferation was inhibited by the concurrent presence of *S. epidermidis* secretome. The two secretomes also have effects on Treg function. More specific, the secretome of *S. epidermidis* elicited Treg activity favoring the suppression of CD4^+^ T cell activation, whereas when the *S. aureus* secretome was present, Treg did not show this effect. This study supports the involvement of *S. aureus* in the onset and promotion of cutaneous inflammation by inducing Th2 activation and suppressing the resident Treg cells.

Iwamoto et al. [94] reported that *S. aureus* from AD skin was able to change the synthesis of cytokines via monocyte-derived Langerhans cells inducing an imbalanced Th1/Th2 skin immunity. In addition to *S. aureus*, the other microbes constituting the cutaneous microbiome could have an important role in the onset and progression of AD [92].

Song et al. [93] observed a significant correlation between the high abundance of *F. prausnitzii* subspecies in the gut microbiome and AD. This enrichment could lead to a reduction of butyrate and propionate producers and thus a diminishment in these two molecules with anti-inflammatory activity. In particular, among the species producing butyrate and propionate, those related to the strain A2-165 are also affected by the change in composition, and their deficiency has been related to CD.

The Suzuki [97] group has investigated whether an abnormal immune response toward microbial stimuli derived via the colonization of beneficial bacteria could be implicated in AD onset. They reported that the stimulation with heat-killed Gram-positive bacteria (*Bifidobacterium bifidum* and *Lactobacillus rhamnosus GG*) and *Lactobacillus*-derived peptidoglycan of cord-blood mononuclear cells (CBMCs) derived from AD infants produced a lower synthesis of IL-10 with respect to infants without AD. This finding has suggested a putative correlation between these bacteria and a higher risk of infantile AD.

AD pathogenesis is not only due to microbiome, but also to extracellular vesicles (EVs) released from bacteria and containing pathogenic proteins from *S. aureus*. These have been correlated with AD onset as demonstrated by in vitro and in vivo studies performed by Hong et al. [89]. More in detail, *S. aureus* EVs were able to promote inflammatory responses via dermal fibroblasts and the thickening of the epidermis associated with the infiltration of mast cells and eosinophils when EVs were applied to tape-stripped mouse skin [89]. EVs derived from the microbiome have been identified in the blood [89], as well as in other organ systems to promote inflammation [187]. AD subjects presented meaningfully elevated serum levels of *S. aureus* EV-specific IgE respect to healthy subjects [89].

As demonstrated recently by Kim et al. [96] on 27 AD patients and 6 healthy controls, the bacterial EV composition differs significantly between the two groups evaluated, with a marked reduction of *Lactococcus*, *Leuconostoc*, and *Lactobacillus* EV proportions and an increase of those from *Alicyclobacillus* and *Propionibacterium* in AD patients with respect to controls. In addition, EVs produced from lactic acid bacteria exerted a protective function against the *S. aureus* EV-induced AD mouse model. Also, Francuzik et al. [95] recently observed differences between lesional and non-lesional skin in AD patients, where the latter showed a diminished abundance of *Propionibacterium* (*P.*) *acnes* and *Lawsonella clevelandensis* and an increase of *S. aureus*. The observation that the products of fermentation of *P. acnes* blocked *S. aureus* and *S. epidermidis* growth, as well as serum collected from AD patients halted *S. aureus* growth more efficiently than serum from healthy individuals, allowed the researchers to hypothesize that specific changes in the cutaneous microbiota could represent a possible strategy for AD treatment.

### 2.7. Autoimmune Neurological Diseases

The incidence of autoimmune neurological diseases, including multiple sclerosis (MS), as well as other autoimmune disorders, has dramatically increased in industrialized Western countries [136,188]. It has been supposed that the diet present in these societies, with rich fat content and reduced intake of fibers, as opposite to societies characterized by a traditional lifestyle [53,189], could induce changes in the composition of the gut microbiome and its activities promoting the onset of autoimmune conditions [190,191].

The intake of fiber through the diet are important for the health of humans since they exert several physiological effects, as modulation of the gut immunological microenvironment of the gut and the protection against autoimmune and allergic diseases induced by short chain fatty acids (SCFAs) representing the final-products of the fiber fermentation [53,189,190,191,192,193]. The role of insoluble fibers, including cellulose, in the etiology of autoimmune conditions remains to be elucidated, though they are able to shape the diversity of the microbiome [192,193]. MS onset has been linked to dietary exposure [98,194,195]. Berer et al. [105] observed that autoimmune demyelination was promoted by auto-reactive B lymphocytes induced after the stimulation of the commensal microbiome with the autoantigen myelin oligodendrocyte glycoprotein (MOG). Conversely, CNS inflammation in mice was contrasted with the expansion of CD4^+^ Foxp3^+^ Tregs via TLR2-mediated CD39 signaling upon the administration of polysaccharide A (PSA), an intestinal commensal product derived from *B. fragilis* [99,100]. Other microbial metabolites able to reduce neuroinflammation are those derived from dietary tryptophan (Trp), which is introduced with the diet and metabolized by the commensal gut microbiome [196] into several AHR agonists and exerts its activity on astrocytes [104]. In association with Trp-derived metabolites, type I interferon (IFN-I) is also synthesized in the CNS function and the axis IFN-I/AhR is involved in the regulation of astrocyte functions and the inflammatory process affecting the CNS [104]. However, MS patients presented lower levels of circulating AHR agonists and a reduced AhR agonistic activity than controls in order to hypothesize the involvement of the commensal microbiome metabolism, diet, or the environment in MS pathogenesis [104].

Furthermore, recent studies have highlighted the presence of dysbiosis in the gut microbiome of patients with MS [101,102,103]. Miyake et al. [101] observed a considerable reduction of species belonging to *Clostridia XIVa* and *IV Clusters*. The study conducted by Jangi et al. [103] on 60 MS patients and 43 healthy controls revealed gut microbiome alterations which included an increased abundance of *Methanobrevibacter* and *Akkermansia* and a reduction in *Butyricimonas*. These defects were associated with an altered expression of genes playing a role in dendritic cell maturation, interferon signaling and nuclear factor-κB (NF-κB) signaling pathways in circulating T lymphocytes and monocytes. In addition, a decrease in *Sarcina* and a rise in *Prevotella* and *Sutterella* occurred in patients on disease-modifying treatment respect to untreated patients [103]. The presence of dysbiosis involving *Akkermansia* (*A.*) *muciniphila* has also been confirmed recently by Cekanaviciute et al. [106] who reported an enhancement in *A. muciniphila* and *Acinetobacter* (*A.*) *calcoaceticus* in MS patients associated with a reduction in *Parabacteroides* (*P.*) *distasonis*. The exposure of human peripheral blood mononuclear cells and monocolonized mice to *A. muciniphila* and *A. calcoaceticus* individual bacterial cultures shifted T lymphocytes towards a pro-inflammatory phenotype. A reduction in Treg compartment associated with a higher number of effector CD4^+^ lymphocytes that differentiated into IFNγ-producing Th1 cells were induced by *A. calcoaceticus*. Th1 lymphocyte differentiation was even more marked upon exposure to *A. muciniphila*. Conversely, *P. distasonis* induced anti-inflammatory IL-10-expressing human CD4^+^ CD25^+^ T cells and IL-10^+^ FoxP3^+^ Tregs in mice [106].

The group of Chen [102] reported an enhancement of *Pseudomonas*, *Mycoplana*, *Haemophilus*, *Blautia*, and *Dorea* in relapsing remitting MS (RRMS) (*n* = 31) patients, while *Parabacteroides*, *Adlercreutzia* and *Prevotella* genera were increased in healthy controls (*n* = 36).

Recently, the groups of Berer [105] and Cekanaviciute [106] demonstrated that microbiome transplantation from MS patients into germ-free mice enables spontaneous experimental autoimmune encephalomyelitis (EAE) in mice [105,106] and a IL-10^+^ Treg subset was lowered with respect to mice transplanted with microbiota from healthy controls [106].

The recent study conducted by Berer et al. [105] found that spontaneous CNS-directed autoimmunity onset is suppressed by a diet with a crude high non-fermentable fiber content (26% of cellulose content, cellulose rich (CR)) during early adult life. In detail, they use genetically engineered spontaneous experimental opticospinal encephalomyelitis (OSE) mice as a spontaneous model for EAE [197] with respect to classic active EAE models such that microbiota composition and immune responses could not be exogenously biased by the use of adjuvants [197]. In the control diet-fed mice (standard rodent diet), spontaneous EAE (sEAE) incidence was about 55%, whereas EAE incidence was strongly decreased (23%) when a CR diet was used, and moreover, OSE mice showed delayed neurological symptom onset. Disease severity, as well as inflammatory marker expression in the spinal cord in EAE mice, did not show any differences. The investigation of the cytokine expression profile revealed that CR diet was correlated with a reduction in the pathogenic T cell response with respect to control diet-fed animals, and in addition, T lymphocytes from mice fed with a CR diet had higher transcript levels of the Th2 cell-associated cytokines than T cells from control mice. The characterization of the cecal microbiota of CR diet and control diet-fed revealed that dietary regimens skewed gut microbial and metabolic profiles. More specifically, OSE mice fed with a CR diet presented an increase of the genera *Helicobacter*, *Enterococcus*, *Desulfovibrio*, *Parabacteroides*, *Pseudoflavonifractor*, and *Oscillibacter*, whereas *Lactobacillus*, *Parasutterella*, *Coprobacillus*, and *TM7 genera Incertae Sedis* were considerably diminished with respect to the control group. It has been hypothesized that the increased Th2 cell response was promoted in CR diet-fed mice and thus the protective effect against EAE onset could have been a consequence of the modified gut microbiota and/or the altered metabolic profile [198].

## 3. Conclusions

In the last few years, autoimmune and inflammatory disorder incidence has considerably increased worldwide, and increasing observations have reported a correlation between the presence of microbiome dysbiosis and the development of different autoimmune conditions, although the precise mechanism remains to be elucidated. Furthermore, limited knowledge is currently available on whether these modifications in microbiome composition could be causally related to the pathogenesis of autoimmunity or these alterations could be a consequence of an abnormal immune response. However, since the gut microbiome is able to shape the host adaptive immune responses through mediator and nutrient release, and moreover dysbiosis of specific human gut bacteria has been found in several different autoimmune conditions, the manipulation of the microbiome could represent a potential therapeutic strategy for the improvement and potentially complete restoration of the normal immune response in different autoimmune diseases.

As emphasized above, diet has the strongest influence on gut microbiota [137]. Nevertheless, to date, few clinical studies of dietary interventions on human gut microbiota have been reported [137]. Definitively, a healthy status is associated with a low energy and high fiber and vegetables intake. In the future, perspectives of human health can certainly be derived from diet regimen control together with synbiotic administration of microbial taxa in order to equilibrate gut microbiota composition [189,192,193,199,200,201,202]. Regarding in particular the focus of the present review, several studies pointed to the selection of microbial species that could improve the treatment of chronic inflammatory disorders in addition to other conditions, including atherosclerosis, behavior abnormalities, cancer, *Clostridium difficile* infection, and obesity. Indeed, Treg expansion was promoted by certain gut bacterial species [137,138]. In mouse models of colitis and allergic inflammation, *Lactococcus lactis*-expressing IL-10 treated inflammation [203] and was safe when administered in a human phase I trial [204]. *E. coli*-secreted proteins were shown to activate anorexigenic pathways to control satiety for obesity treatment [205]. Further in diabetic rats, metabolism control was ameliorated using glucagon-like peptide 1-releasing bacterial strains with the effect of inducing insulin secretion [206].

A future avenue for treatment is fecal microbiota transplantation (FMT). Studies have already documented FMT as a medically actionable tool, for example, in treating recurrent diarrhea using *Clostridium difficile* or insulin-resistance in obese patients [207]. This evidence could foster future application studies aimed to control inflammation in patients affected by autoimmunity.

## Figures and Tables

**Table 1:** Microbiome alterations in different autoimmune conditions respect to healthy subjects.

Autoimmune Conditions	Microbiome Alterations in Autoimmunity Respect to Healthy Subjects	References
**Type I diabetes**	↓ bacterial diversity in high-risk children	[35,42,43]
	↑ *Bacteroidetes*/*Firmicutes* ratio	[36,44,51]
	↑ *Bacteroidetes, Clostridium*, and *Veillonella*; ↓ *Bifidobacterium*, *Lactobacillus*, *Blautia coccoides*/*Eubacterium rectale* group, and *Prevotella*	[37]
	↑ *Bacteroides dorei* in high-risk children	[39]
	↑ *Bacteroides* and ↓ *Prevotella* in newly diagnosed T1D patients	[40]
	↓ *Bifidobacterium*	[36,41]
	↑ *Bacteroides* and *Clostridium* cluster XVa and cluster IV; ↓ *Bifidobacterium*	[47]
	↑ *Candida albicans* and *Enterobacteriaceae*	[41]
**Rheumatoid arthritis**	↑ of the pathobiont *Prevotella (Prevotella copri)* in new-onset RA subjects	[107]
	Gut and oral microbiome dysbiosis; ↓ *Haemophilus spp.* and ↑ *Lactobacillus salivarius*	[56]
	↓ gut bacterial diversity and expansion of rare lineage intestinal microbes	[57]
	Association between periodontal infection due to *Porphyromonas gingivalis* and RA	[54,108]
	↑ *Fretibacterium, Selenomonas* and *Prevotella nigrescens*	[55]
	↑ *Bacilli* and *Lactobacillales*; ↓ genus *Faecalibacterium* and the specie *Faecalibacterium prausnitzii*; Absence of the genus *Flavobacterium* and the species *Blautia coccoides* in RA patients present instead in controls	[62]
	↑ *Prevotella copri* and ↓ *Bacteroides* in new-onset untreated RA patients	[107]
**Systemic lupus erythematosus**	↑ *Bacteroidetes*/*Firmicutes* ratio	[65]
	Association between SLE and periodontal disease; Dysbiosis of the subgingival microbiota; ↑ subgingival bacterial load; ↓ subgingival microbial diversity at diseased sites	[67]
	↑ *Fretibacterium*, *Selenomonas*, and *Prevotella nigrescens*;	[109]
	Association with periodontal pathogens (*Treponema denticola*, *Porphyromonas gingivalis*, *Fretibacterium fastidiosum* and *Tannerella forsythia*	[109,110]
**Behcet’s disease**	↓ *Roseburia* and *Subdoligranulum* genera	[79]
	↑ *Bifidobacterium* and *Eggerthella* genera and ↓ *Megamonas* and *Prevotella* genera	[81]
	↑ *Bilophila spp.* and several opportunistic pathogens (e.g., *Parabacteroides spp.* and *Paraprevotella spp.*); ↓ butyrate-producing bacteria *Clostridium spp.* and methanogens (*Methanoculleus spp.* and *Methanomethylophilus spp.*).	[82]
**Inflammatory bowel disease**	↓ gut bacterial diversity	[68]
	↓ diversity in the bacterial phylum *Firmicutes faecal*; ↓ *Clostridium leptum* phylogenetic group	[70]
	↑ *Proteobacteria* phylum including Escherichia *coli*; ↓ *Firmicutes* phylum was reduced	[69,70,73,111]
	↓ *Faecalibacterium prausnitzii* is associated with an ↑ risk of postoperative recurrence of ileal CD	[72]
	↓ in several butyrate-producing bacteria species	[74,76]
	↓ of the genera *Bacteroides*, *Eubacterium*, *Faecalibacterium* and *Ruminococcus*; ↑ genera *Actinomyces* and *Bifidobacterium*; ↓ butyrate-producing bacterial species, as *Blautia faecis*, *Roseburia inulinivorans*, *Ruminococcus torques*, *Clostridium lavalense*, *Bacteroides uniformis*, and *Faecalibacterium prausnitzii*	[76]
	Dysbiosis	[112]
	↑ *Caudovirales bacteriophages* and fungal composition	[113]
**Vitiligo**	↑ *Actinobacteria*, *Proteobacteria*, *Firmicutes*, and *Bacteroidetes*	[83,114]
	↓ bacterial diversity	[83]
**Psoriasis vulgaris**	↑ *Proteobacteria*; ↓ *Staphylococci* and *Propionibacteria*	[85]
	↓ bacterial diversity; ↑ *Corynebacterium*, *Propionibacterium*, *Staphylococcus*, and *Streptococcus*; ↓ *Cupriavidus*, *Flavisolibacter*, *Methylobacterium*, and *Schlegelella* genera. Association between lesion samples with *Firmicutes*-associated microbiota	[86]
	↓ diversity and ↑ *Staphylococcus* in psoriatic ear sites	[87]
	↑ diversity and ↑ heterogeneity ↑ *Staphylococcus aureus*; ↓ *Staphylococcus epidermidis* and *Propionibacterium acnes*	[88]
**Atopic dermatitis**	↑ *Faecalibacterium prausnitzii* subspecies	[93]
	↑ *Staphylococcus aureus*	[94]
	↓ *Propionibacterium acnes* and *Lawsonella clevelandensis*; ↑ *Staphylococcus aureus* in non-lesional relative to lesional AD patients	[95]
**Autoimmune neurological diseases**	↓ species belonging to *Clostridia XIVa* and *IV Clusters*	[101]
	↑ *Pseudomonas*, *Mycoplana*, *Haemophilus*, *Blautia*, and *Dorea* in relapsing remitting MS patients; ↓ *Parabacteroides*, *Adlercreutzia*, and *Prevotella* genera	[102]
	↑ *Methanobrevibacter* and *Akkermansia*; ↓ *Butyricimonas*	[103]
	↑ *Akkermansia muciniphila* and *Acinetobacter calcoaceticus;* ↓ *Parabacteroides distasonis*	[106]

**Table 2:** Bacterial associated mechanisms promoting autoimmunity.

Autoimmune Disorders	Bacterial Associated Mechanisms Promoting Autoimmunity	References
**Type I diabetes**	Perturbation in the integrity of epithelial barrier	[49,115,116,117,118,119]
	Changes in gut microbiota following antibiotic treatment	[120,121,122]
	Functional enrichment in core energy metabolism proteins, in particular on sugar transport and processing	[47]
	Perturbations in the integrity of epithelial tight junctions	[44]
	Blockage of Treg differentiation via products generated with the anaerobic respiration, such as acetate and succinate	[44]
	Increased intestinal inflammation and reduced barrier activity due to depletion in microbiota taxa related with host proteins implicated in the maintenance of mucous barrier functionality, microvilli adhesion, and exocrine pancreas	[48]
	Reduced presence of beneficial anaerobic gut bacteria *Lactobacillus*, *Bifidobacterium*, and *Bacteroides* species exerting an inhibitory function by synthetizing short-chain fatty acids and antimicrobial compounds	[36,41]
	Bacterial metabolites can affect host immune system leading to pro- or anti-inflammatory reactions	[53]
**Lung autoimmunity in rheumatoid arthritis**	Modulation of host immune response by gut-residing segmented filamentous bacteria via increased Th17cells percentages induced by the strong Th17 chemoattractant CCL20	[123,124,125]
**Rheumatoid arthritis**	Promotion of autoantibodies by gut residing segmented filamentous bacteria during the pre-arthritic phase	[126]
	Molecular mimicry between several gut microbe epitopes and two autoantigens *N*-acetylglucosamine-6-sulfatase and filamin A greatly expressed in inflamed synovial tissue	[58]
	Citrullinated proteins via the peptidylarginine deiminase (PAD) enzyme derived from bacteria	[127,128,129]
**Systemic lupus erythematosus**	Correlation between dysbiotic periodontal inflammation and more severe SLE scores	[67]
**Behcet’s disease**	Altered Th1, Th17, and Treg functions	[130,131]
	Reduction in butyrate-producing bacteria and methanogens, with enhanced oxidation-reduction process, capsular polysaccharide transport system, and type III and IV secretion systems	[82]
	Strong intraocular inflammatory reaction	[82]
**Inflammatory bowel disease**	Impaired epithelial barrier and increased intestinal permeability	[111]
	Cellular stress responses interacting with microbiome in the gastrointestinal tract. Interaction between bacteria and endoplasmic reticulum	[78]
	Reduction of several butyrate-producing bacteria	[74,76]
	Reduced AHR agonists in the inflamed intestinal tissue samples that modulate T cell responses AHR agonists exert an anti-inflammatory effect inducing IL-22	[132,133,134]
**Vitiligo**	Aberrant intra-community network in the lesional skin areas respect to those of non-lesional sites. Dysbiosis of diverse microbial community in vitiligo lesional skin	[135]
**Psoriasis vulgaris**	Increased Th17 response which could have a role in IL-17-driven inflammation	[88]
**Atopic dermatitis**	Increase of IgE response, inflammatory and Th2/Th22 transcripts, promotion of Th2 activation, and suppression of resident Treg cells by secretomes of skin microbiota	[91]
	Induction of an imbalanced Th1/Th2 skin immunity	[94]
	Decrease of butyrate and propionate producers (molecules with an anti-inflammatory activity)	[93]
	EVs derived from the microbiome and increase of inflammation	[89,136]
**Multiple sclerosis**	Reduced levels of circulating AHR agonists and reduced AHR agonistic activity	[104]
	Modulation by fecal microbiota abundance of expression of host immune genes involved in dendritic cell maturation, and interferon and NF-kB signaling pathways in circulating T lymphocytes and monocytes	[103]
	Reduced Treg compartment associated with increased percentages of effector CD4^+^ lymphocytes that differentiated into IFNγ-producing Th1 cells Reduced IL-10^+^ Treg subset in mice transplanted with microbiota from MS patients	[106]
	Diet skewed gut microbial and metabolic profiles	[105,137,138]

## Abbreviations

ACPA	anti–citrullinated protein antibody
AD	atopic dermatitis
AHR	aryl hydrocarbon receptor
BD	Behcet’s disease
BPB	butyrate-producing bacteria
CBMCs	cord-blood mononuclear cells
CCL20	chemokine (C-C motif) ligand 20
CD	Crohn’s disease
CIA	collagen-induced arthritis
CNS	central nervous system
CR	cellulose rich
CS	caesarian section
EAE	autoimmune encephalomyelitis
EVs	extracellular vesicles
FLNA	filamin A
FMT	fecal microbiota transplantation
GNS	*N*-acetylglucosamine-6-sulfatase
HbA1c	hemoglobin A1c
HCD	hydrolyzed casein diet
IBD	inflammatory bowel disease
IFN-I	type I interferon
IFN-γ	interferon gamma
IL	Interleukin
LPS	lipopolysaccharide
MOG	myelin oligodendrocyte glycoprotein
MS	multiple sclerosis
NF-κB	nuclear factor-κB
NOD	non-obese diabetic mice
OSE	opticospinal encephalomyelitis
PAD	peptidylarginine deiminase
PAMPs	pathogen associated molecular patterns
PSA	polysaccharide A
RA	rheumatoid arthritis
RRMS	relapsing remitting MS
SCFAs	short-chain fatty acids
SCFAs	short chain fatty acids
sEAE	spontaneous EAE
SFB	segmented filamentous bacteria
SLE	systemic lupus erythematosus
SPF	specific-pathogen-free
T1D	type I diabetes
Tc	cytotoxic T lymphocytes
TCRs	T cell receptors
Tfh	T follicular helper
Th	T lymphocytes. T-helper
Treg	regulatory T
Trp	tryptophan
UCs	ulcerative colitis
